# C/EBPβ isoform-specific regulation of migration and invasion in triple-negative breast cancer cells

**DOI:** 10.1038/s41523-021-00372-z

**Published:** 2022-01-18

**Authors:** Britt A. Sterken, Tobias Ackermann, Christine Müller, Hidde R. Zuidhof, Gertrud Kortman, Alejandra Hernandez-Segura, Mathilde Broekhuis, Diana Spierings, Victor Guryev, Cornelis F. Calkhoven

**Affiliations:** 1 European Research Institute for the Biology of Ageing (ERIBA), University Medical Center Groningen, University of Groningen, Antonius Deusinglaan 1, Groningen, 9700 AD The Netherlands; 2 iPSC CRISPR facility, University Medical Center Groningen, Groningen, The Netherlands; 3 Research Sequencing facility, University Medical Center Groningen, Groningen, The Netherlands

**Keywords:** Oncogenes, Breast cancer

## Abstract

The transcription factor C/EBPβ is a master regulator of mammary gland development and tissue remodelling during lactation. The *CEBPB*-mRNA is translated into three distinct protein isoforms named C/EBPβ-LAP1, -LAP2 and -LIP that are functionally different. The smaller isoform LIP lacks the N-terminal transactivation domains and is considered to act as an inhibitor of the transactivating LAP1/2 isoforms by competitive binding for the same DNA recognition sequences. Aberrantly high expression of LIP is associated with mammary epithelial proliferation and is found in grade III, estrogen receptor (ER) and progesterone (PR) receptor-negative human breast cancer. Here, we show that reverting the high LIP/LAP ratios in triple-negative breast cancer (TNBC) cell lines into low LIP/LAP ratios by overexpression of LAP reduces migration and matrix invasion of these TNBC cells. In addition, in untransformed MCF10A human mammary epithelial cells overexpression of LIP stimulates migration. Knockout of *CEBPB* in TNBC cells where LIP expression prevails, resulted in strongly reduced migration that was accompanied by a downregulation of genes involved in cell migration, extracellular matrix production and cytoskeletal remodelling, many of which are epithelial to mesenchymal transition (EMT) marker genes. Together, this study suggests that the LIP/LAP ratio is involved in regulating breast cancer cell migration and invasion. This study together with studies from others shows that understanding the functions the C/EBPβ-isoforms in breast cancer development may reveal new avenues of treatment.

## Introduction

The CCAAT/enhancer-binding protein family of basic region leucine-zipper (bZIP) transcription factors consists of six members (designated α to ζ) that are widely expressed and have functions involved in cell proliferation, differentiation, metabolism, and senescence^1,2^. C/EBPs regulate gene transcription through the formation of dimers and binding to C/EBP-specific recognition sites in the genome. Expression of C/EBPβ as well as of the related C/EBPα proteins is uniquely regulated at the level of mRNA translation involving a *cis*-regulatory upstream open reading frame (uORF) and translation into three protein isoforms of different length from a single mRNA molecule^3^. The C/EBPβ protein isoforms LAP1, LAP2 (Liver-enriched Activating Protein; also called LAP* and LAP) originate through regular translation initiation, and they are transcriptional activators. From the same mRNA an additional isoform LIP (liver-enriched inhibitory protein) is translated from a downstream AUG codon, omitting the N-terminal transactivation domains. LIP is generally considered a competitive inhibitor of LAP, although some studies suggest LIP has transactivating functions in addition^4–6^. All isoforms share a highly conserved C-terminal bZIP domain containing a basic DNA-interaction region and a leucine zipper dimerization domain. Expression of LIP requires initial translation of the uORF followed by a translation re-initiation event at the LIP-AUG codon^3,7^ (Supplementary Fig. 1a). The efficiency of LIP expression is regulated by mTORC1 signalling and prospectively through other pathways yet to be identified^8^. Differential expression of LAP and LIP is involved in breast tissue development, tissue remodelling during pregnancy and lactation, with LIP expression being elevated in proliferative phases^9–14^. Genetic ablation of the *Cebpb* gene in mouse mammary glands results in defective mammary ductal morphogenesis, lobuloalveolar development and differentiation of mammary epithelial cells^13^. In addition, using *Cebpb*-knockout models it was shown that C/EBPβ is required for the expansion of mammary stem cells and for regular luminal cell lineage commitment^15^. Low LIP/LAP expression ratios are observed in untransformed cells or tissues surrounding the breast tumour^16^. In contrast, much higher expression of LIP compared to LAP has been clearly associated with high histological carcinoma grading (poorly differentiated) and highly proliferative (high Ki67 staining) aggressive tumours, including the oestrogen receptor-negative (ER−), progesterone receptor-negative (PR−) tumours^16–18^. In addition, a high LIP/LAP expression ratio was linked to metastatic breast cancer samples that were defective in the TGFβ-induced cytostatic response, whereas a low LIP/LAP ratio was associated with an intact growth-inhibitory TGFβ response. The TGFβ cytostatic response in cells with a high LIP/LAP ratio could be restored by re-expression of LAP. Since evasion of the TGFβ cytostatic response is a critical step during breast cancer development towards a more migratory and invasive metastatic phenotype^19^ these data suggest that C/EBPβ isoform de-regulation (towards a higher LIP/LAP ratio) is crucially involved in promoting breast cancer metastasis^20^.

The shift in high LIP/LAP expression instead of a general higher expression with preserved isoform ratio suggest that the underlying mechanism is post-transcriptional. In line with a translational mechanism is the finding that in HER2-overexpressing breast cancer cells HER2 activates the RNA-binding factor CUGBP1, which stimulates LIP expression over that of LAP^21^. Studies with genetically modified mouse models support that high LIP/LAP expression ratios are linked to the development of breast cancer. Specific overexpression of LIP in the mammary epithelium induces hyperplasia and neoplasia, although the latter with low frequency^9^. Mice that are systemically deficient in LIP expression but proficient in LAP expression, through mutation of the uORF in the *Cebpb* gene, show an overall reduced tumour incidence^8,22^. Conversely, systemic mono- or biallelic ablation of LAP with retainment of LIP increases tumour incidence in mice^23^. Altogether, the studies demonstrate that aberrant translation of the *CEBPB*-mRNA into its different protein isoforms plays a crucial role in breast cancer development, although we do not fully understand the underlying complexity^24^.

Here, we show that triple-negative breast cancer (TNBC) cell lines express C/EBPβ with high LIP to LAP isoform ratios in contrast to luminal A breast cancer cell lines or untransformed mammary epithelial cells that express low LIP to LAP ratios. Reversing C/EBPβ expression to low LIP/LAP ratios in TNBC cell lines by overexpression of LAP reduces the migration and invasion potential of these cancer cells, whereas overexpression of LIP stimulates the migration in untransformed epithelial cells. Using CRISPR/Cas9 mediated knockout of *CEBPB* and genome-wide RNA-sequencing we reveal that expression of genes associated with epithelial to mesenchymal transition (EMT) and the extracellular matrix (ECM) partially depend on LIP and/or LAP expression. Altogether, our data suggest a role for high LIP/LAP expression ratio in the regulation of breast cancer cell migration and ECM remodelling, two key characteristics that are associated with the aggressive phenotype of TNBC.

## Results

### TNBC cell lines express C/EBPβ with high LIP to LAP isoform ratios

**Fig. 1:**
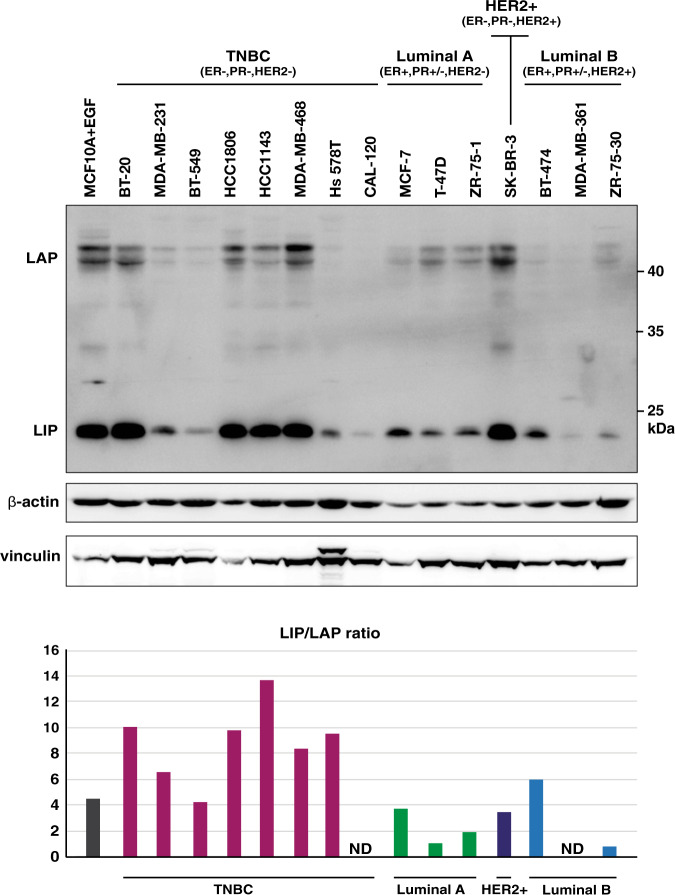
High LIP/LAP expression ratios in TNBC cells. The immunoblot shows the expression of LIP and LAP in a panel of TNBC (ER−, PR−, HER2−), luminal A (ER+, PR+/−, HER2−), HER2-positive (+) (ER−, PR−, HER2+), luminal B (ER+, PR+/−, HER2+) type breast cancer cell lines^25^ and in untransformed MCF10A supplemented with EGF required for proliferation and known to induced LIP. The blots below show β-actin as a low-molecular and vinculin as a high-molecular loading control. The bar graph shows the relative LIP/LAP isoform ratio quantification per cell line of the blot shown. The LAP signals of CAL-120 and MDA-MB-361 were too low for reliable quantification.

We analysed C/EBPβ expression in the TNBC (ER−, PR−, HER2−) derived cell lines BT-20, MDA-MB-231, BT-549, HCC1806, MDA-MB-468, Hs 578T and CAL-120, the luminal A derived (ER+, PR+, HER2−) cell lines MCF-7, T-47D and ZR-75-1, the HER2-positive derived cell line (ER−, PR−, HER2+) SK-BR-3, and the luminal B derived (ER+, PR+, HER2+) cell lines BT-474, MDA-MB-361 and ZR-75-30^25^ as well as in the untransformed mammary epithelial cell line MCF10A cultured in media supplemented with EGF that is known to stimulate LIP expression through CUGBP1 activation^26^. The immunoblots and the bar graphs of the signal quantifications show that LIP/LAP isoform ratios are high in TNBC cells, most evidently in the cell lines with overall high expression of LIP and LAP. The luminal A cells show lower LIP/LAP rations with lower overall LIP and LAP expression. In addition, LIP/LAP isoform ratios are moderately high in HER2-positive cells as has been shown before^21^ with variable overall expression levels. The MCF10A cells also express moderately high LIP/LAP ratio (Fig. 1 and Supplementary Fig. 1b for additional independent data). Thus, similar to what was observed in patient samples of hormone receptor-negative breast cancer^17,18^, TNBC cells are characterized by high levels of LIP and high LIP/LAP expression ratios.

To examine whether the presence of the estrogen receptor (ER) or progesterone receptor (PR) in the luminal A-derived cancer cell lines affects LIP/LAP expression we cultivated MCF-7 cells in hormone-depleted medium and treated the cells with estrogen (E) or progesterone (P) and did not observe significant changes in the LIP/LAP isoform ratio (Supplementary Fig. 1b). This indicates that the difference in the LIP/LAP ratio between the hormone receptor-positive luminal A-derived and the hormone receptor-negative TNBC-derived cell lines is not due to the different ER or PR expression status.

### C/EBPβ regulates migration- and microenvironment-related genes in TNBC cells

**Fig. 2:**
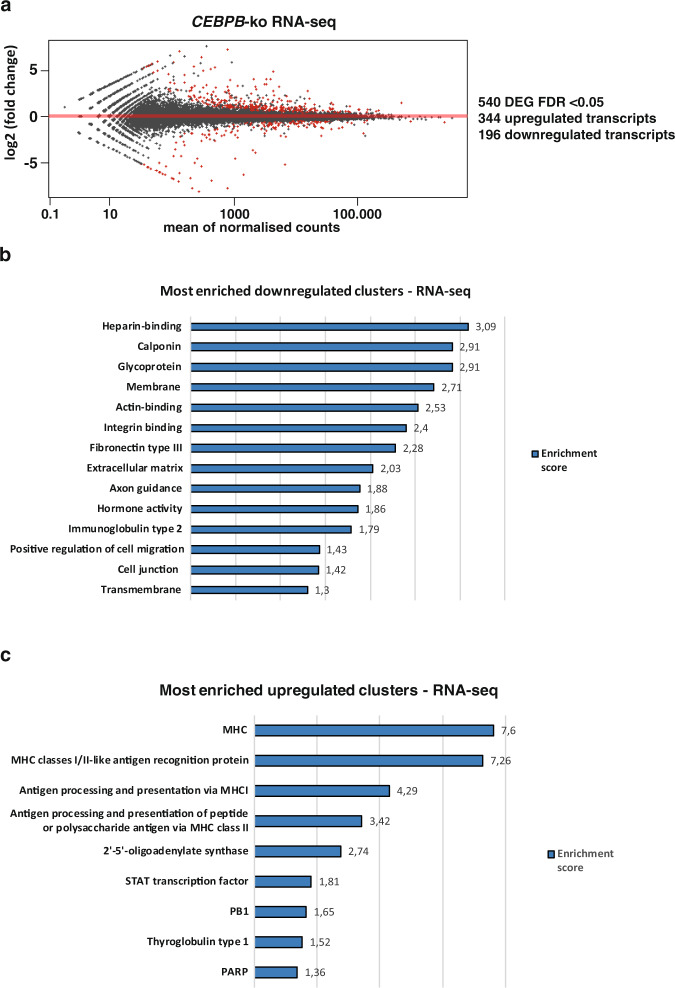
Transcriptomics and functional clustering analysis of BT-20 *CEBPB*-ko vs BT-20 wt cells. **a** Differentially expressed genes (DEG) in BT-20 *CEBPB*-ko cells vs BT-20 wt cells with an FDR < 0.05. **b** Most downregulated clusters (DAVID) of mRNAs downregulated in *CEBPB*-ko cells (medium stringency). **c** Most upregulated clusters (DAVID) of mRNAs upregulated in *CEBPB-*ko cells (high stringency).

To study the downstream transcriptional effects of the high LIP/LAP isoform ratio, we analysed the transcriptional changes upon CRISPR/Cas9 mediated knockout (ko) of *CEBPB* in the TNBC cell line BT-20 by genome-wide RNA sequencing (RNA-seq) (Fig. 2a, Supplementary Fig. 2a and b and Supplementary Data 1). Differential expression analysis using the the R package DESeq2^27^ revealed upregulation of 344 genes and downregulation of 196 genes in the *CEBPB*-ko BT-20 cells vs BT-20 wt cells (Fig. 2a and Supplementary Data 1). That we found more genes upregulated is congruent with the loss of the predominantly expressed transcriptional repressor LIP in BT-20 cells. Grouping of the differentially expressed genes using the DAVID Functional Clustering tool (https://david-d.ncifcrf.gov/) revealed downregulated clusters involved in cell migration, actin binding and ECM remodelling (Fig. 2b). The most prominent upregulated gene clusters are antigen presentation and MHC I and MHC II expression (Fig. 2c). In a previous study we have performed transcriptome analyses using mouse embryonic fibroblasts (MEFs) derived from *Cebpb*-ko mice, which were reconstituted with LIP expression^28^. In this case, DAVID functional clustering analysis revealed that LIP overexpression causes downregulation of clusters involved in ECM-cell interaction, cell adhesion molecules and collagen subtypes (Supplementary Fig. 2c)^28^. Therefore, we hypothesised that the C/EBPβ isoform ratio might regulate cell migration and ECM-cell interaction.

### C/EBPβ regulates cell migration of TNBC cells

**Fig. 3:**
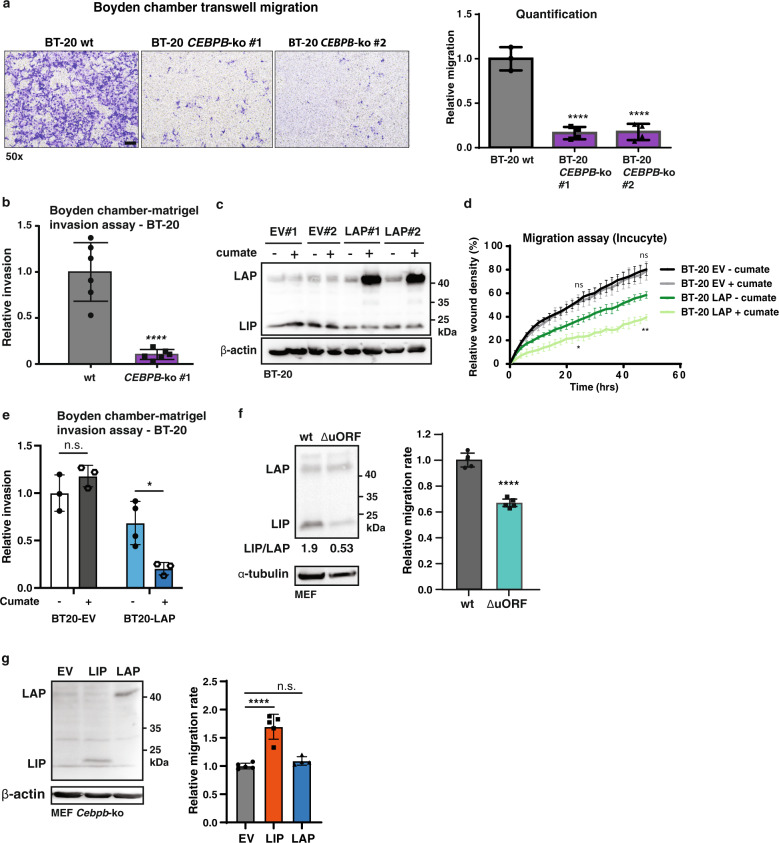
LIP and LAP differentially regulate cell migration and invasion. **a** Representative images of Boyden chamber migration assay of BT-20 wt and two cellular clones of *CEBPB*-ko cells with at the right a bar graph of corresponding quantifications (wt, *n* = 3; *CEBP*-ko *n* = 4). One-way ANOVA analysis; error bars represent SD, *p < 0.05, **p < 0.01, ***p < 0. 001, ****p < 0.0001. scale bar represents 100 μm. **b** Bar graph with representative quantification of 3D Boyden chamber-matrigel invasion assay using BT-20 wt and BT-20 *CEBPB*-ko clone #1 (BT-20 wt, *n* = 6; and BT-20 *CEBPB*-ko, *n* = 6). Statistical differences were analysed after 48 h using an unpaired T-test. Error bars represent SD, *p < 0.05, **p < 0.01, ***p < 0. 001, ****p < 0.0001. **c** Immunoblot showing the expression of LAP and LIP in BT-20 cells containing a cumate-inducible LAP construct or empty vector control (EV), two cellular clones of each. β-actin was used for loading control. **d** Time course of Incucyte scratch wound migration assay shown as relative wound density of BT-20 cells containing a cumate-inducible LAP construct (clone #1) or empty vector control (EV, clone #1) induced with cumate (*n* = 4). Statistical differences were analysed for timepoints 24 and 48 h using Two-way Anova analysis and Tukey’s multiple comparisons test (BT-20 EV− cumate vs BT-20 EV+ cumate were compared (ns), and BT-20 LAP− cumate vs BT-20 LAP+ cumate were compared (**)). Error bars represent SD, *p < 0.05, **p < 0.01, ***p < 0. 001. **e** Bar graph shows quantification of 3D Boyden chamber-matrigel invasion assay using BT-20 cells containing a cumate-inducible LAP construct (clone #1) or empty vector control (EV, clone #1) (BT-20 EV, *n* = 3; BT-20 LAP, *n* = 4). Statistical differences were analysed after 48 h using two-way Anova analysis. Error bars represent SD, *p < 0.05, **p < 0.01, ***p < 0.001. **f** Immunoblot shows the expression of LAP and LIP in wt MEFs and LIP-deficient *Cebpb*^*ΔuORF*^ MEFs with the respective LIP/LAP ratios shown underneath. α-tubulin was used for loading control. The bar graph shows relative migration rate of a scratch wound assay (*n* = 5). Statistical differences were analysed by T-test. Error bars represent SD, *p < 0.05, **p < 0.01, ***p < 0.001, ****p < 0.0001. **g** Immunoblot shows the re-expression of LAP and LIP in *Cebpb*-ko MEFs as well as empty vector control (EV). β-actin was used for loading control. The bar graph shows relative migration rate of a scratch wound assay (*n* = 5). Statistical differences were analysed by T-test. *T* Error bars represent SD, *p < 0.05, **p < 0.01, ***p < 0.001, ****p < 0.0001. The immunoblots from (**f**) and (**g**) are taken from from Ackermann et al.^28^.

**Fig. 4:**
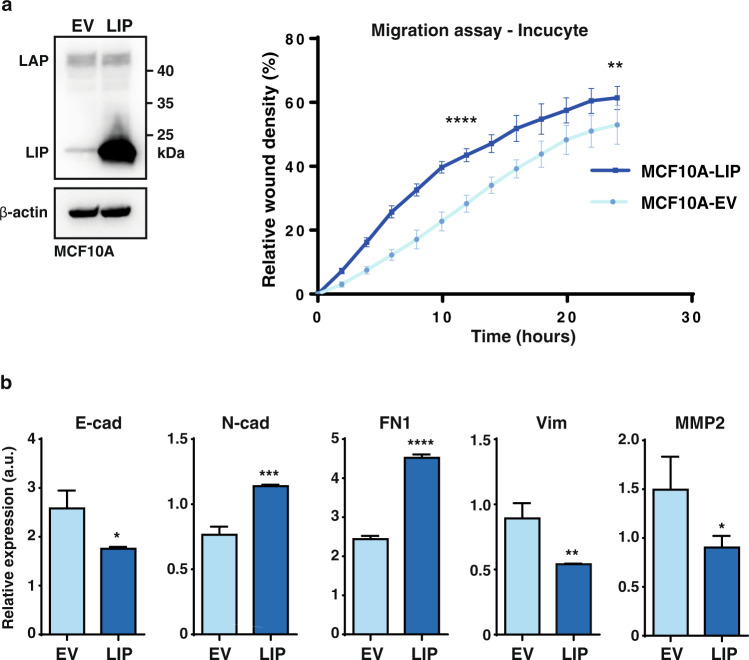
C/EBPβ regulates a subset of EMT markers. **a** Immunoblot showing the expression of LAP and LIP in MCF10A cells containing an expression vector for LIP or empty vector control (EV). β-actin was used for loading control. At the right, a time course of Incucyte migration assay is shown as relative wound closure density of MCF10A cells containing a LIP expression construct or empty vector control (EV) (*n* = 8). Statistical differences were analysed for timepoints 12 h and 24 h using a -T-test. **b** Expression of epithelial and mesenchymal genes relative to GAPDH in MCF10A cells containing a LIP expression construct or empty vector control (EV) (*n* = 3). Results are analysed using a T-test. Error bars represent SD, *p < 0.05, **p < 0.01, ***p < 0.001, ****p < 0.0001.

The observed downregulation of cell migration-related genes in BT-20 *CEBPB*-ko cells motivated us to investigate the effects of C/EBPβ isoform expression on cell migration. Using the Boyden chamber migration assay we examined the migration of *CEBPB*-ko cells versus *CEBPB*-wt cells and observed a decrease in migration of *CEBPB*-ko cells after 48 h (Fig. 3a). In addition, a cell invasion assay using a 3D Boyden chamber with matrigel overlay showed a reduction in cellular invasion for the *CEBPB*-ko cells (Fig. 3b). To verify that LIP but not LAP drives migration, a cumate-inducible LAP construct was introduced in wt BT-20 cells to revert C/EBPβ expression from high LIP/LAP to low LIP/LAP ratios (Fig. 3c). An IncuCyte (Sartorius) life cell imaging scratch wound assay showed that cumate-induced LAP expression results in a significantly reduced migration rate (Fig. 3d). Due to some leaky induction of LAP in the absence of cumate (Fig. 3c; compare - cumate LAP vs. EV) the cumate-uninduced LAP cells show intermediate decrease in cell migration compared to EV and cumate-induced LAP cells (Fig. 3d). A cell invasion assay using a 3D Boyden chamber with matrigel overlay showed that LAP induction in BT-20 cells reduced cell invasion (Fig. 3e and Supplementary Fig. 3a). Similar results were obtained using the BT-549 TNBC cell line with introduced cumate-inducible LAP expression. Induction of LAP expression resulted in reduced cell migration using the Incucyte system (Supplementary Fig. 3b and c). As mentioned above transcriptome analysis showed that expression of LIP in *Cebpb*-ko MEFs affects genes involved ECM-cell interaction and cell migration (Supplementary Fig. 2c)^28^. We therefore examined the effects of LIP/LAP manipulation on cell migration of MEFs. MEFs derived from mice that are deficient in LIP expression (*Cebpb*^*ΔuORF*^) showed a strongly reduced migration rate in scratch wound assay (Fig. 3f). In addition, re-expression of LIP in *Cebpb*-ko MEFs increased the migration rate while re-expression of LAP showed no effect compared to the empty vector (EV) control (Fig. 3g). Altogether, the data show that LAP inhibits migration and invasion of the TNBC cells BT-20 and BT-549, and that LIP promotes migration in MEFs and in untransformed MCF10A breast epithelial cells (Fig. 4a).

### C/EBPβ regulates a subset of EMT markers

**Fig. 5:**
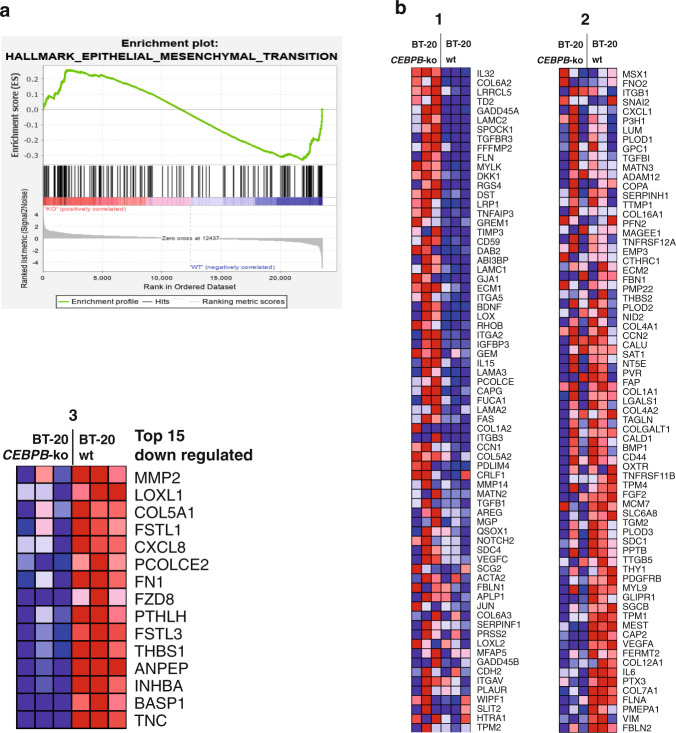
Regulation of ECM genes by C/EBPβ. **a** Gene Set Enrichment Analysis (GSEA) plot of Hallmark Epithelial-Mesenchymal Transition (EMT) gene set. **b** Heatmap of differentially expressed genes from the (EMT) gene set in (**a**) between BT-20 *CEBPB*-ko cells vs BT-20 WT cells split in three parts with in the third (3) enlarged part the top 15 downregulated leading-edge genes.

**Fig. 6:**
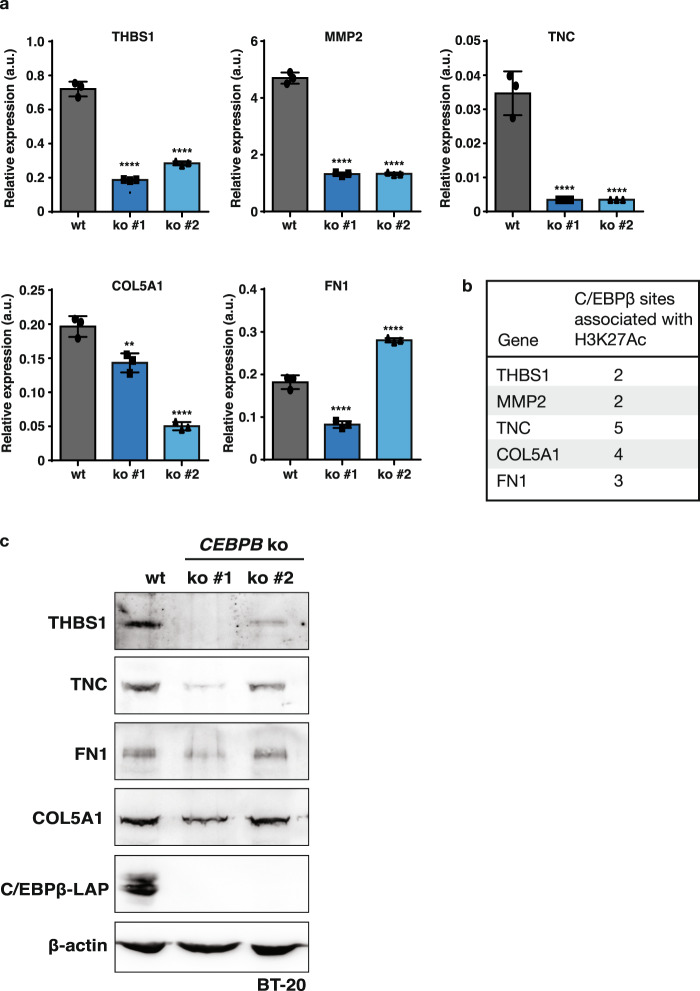
Downregulation of ECM factors in *CEBPB*-ko cells. **a** Expression of ECM-associated genes relative to GAPDH in wt BT-20 cells and two different clones of *CEBPB*-ko BT-20 cells (*n* = 3). Results are analysed using one-way ANOVA analysis. Error bars represent SD, **p < 0.01, ****p < 0.0001. **b** C/EBPβ-associated fragments associated with H3K acetylation (H3K27Ac). Data from the ENCODE database (http://genome.ucsc.edu/ENCODE/)(see Supplementary Table 1). **c** Immunoblots showing expression of Thrombospondin 1 (THBS1), Tenascin C (TNC), Fibronectin 1 (FN1) or Collagen 5A1 (COL5A1) in wt BT-20 cells and two different clones of *Cebpb*-ko BT-20 cells. Loss of C/EBPβ-LAP expression shows the successful *CEBPB*-knockout and β-actin was used for loading control.

In aggressive breast cancer, TNBC in particular, cancer cells show a highly migratory behaviour that is associated with EMT^29,30^. To examine to what extent EMT markers are regulated upon induction of LIP and the associated migratory phenotype, we used the untransformed mammary epithelial cell line MCF10A as the experimental system. MCF10A-LIP overexpressing cells adopt a more spindle-shaped morphology associated with EMT (Supplementary Fig. 4) and show an increase in cell migration as was measured by Incucyte scratch assays (Fig. 4a). Analysis of transcript levels of EMT markers showed that overexpression of LIP resulted in decreased mRNA expression of the epithelial marker and cell adhesion molecule E-cadherin (E-cad), which is characteristic of EMT (Fig. 4b). Also, in line with EMT is the increase in the mesenchymal marker N-cadherin (N-cad) and in the ECM protein Fibronectin (FN1). However, the mesenchymal marker Vimentin (Vim) and the Matrix Metallopeptidase 2 (MMP2) involved in re-organisation of the ECM upon EMT show reduced expression upon LIP overexpression. To analyse to what extent expression of EMT transcripts are altered in the BT-20 cells that predominantly express LIP over LAP versus BT-20 *CEBPB*-ko cells we applied Gene Set Enrichment Analysis (GSEA) (https://www.gsea-msigdb.org/gsea; the Broad Institute) to our transcriptome data. The enrichment plot shows no clear positive or negative correlation in the regulation of the EMT hallmark gene set, but rather a mixed signature of upregulated and downregulated EMT genes (Fig. 5a and Supplementary Data 1). For a more detailed insight, we generated a heat map of EMT genes that were detected in the transcriptome analysis (Fig. 5b). Within the top 15 most downregulated genes upon *CEBPB*-ko within the EMT gene set, a large subset of genes was found to be related to ECM organisation (Fig. 5b). These genes include Tenascin C (TNC), Collagen 5A1 (COL5A1), Fibronectin 1 (FN1), and Thrombospondin 1 (THBS1), and genes related to ECM remodelling, including MMP2, Lysyl Oxidase Like 1 (LOXL1) and Procollagen C-Endopeptidase Enhancer 2 (PCOLCE2), which are all known to contribute to breast tumour progression via increased ECM deposition and remodelling as reviewed in^31^. From these ECM-related genes we chose a subset for verification of the downregulation in two independent BT-20 *CEBPB*-ko clones by RT-qPCR (Fig. 6). Downregulation of the ECM genes THBS1, TNC, COL5A1, MMP2 in response to the *CEBPB*-ko could be confirmed in both knockout clones while a reduction of FN1 expression was detected only in one of the two knock-out clones. The ENCODE database (http://genome.ucsc.edu/ENCODE/) records C/EBPβ-associated fragments associated with H3K acetylation (H3K27Ac) marking transcriptional active regions for all five genes (Fig. 6b). In addition, protein levels of THBS1 and TNC were reduced in the two clones of BT-20 *CEBPB*-ko cells, and protein levels of FN1 and COL5A1 were reduced in one *CEBPB*-ko clone (Fig. 6c). Altogether, the data point to a “partial” EMT regulation by C/EBPβ.

### C/EBPβ-LAP induces the expression of ECM-associated genes

**Fig. 7:**
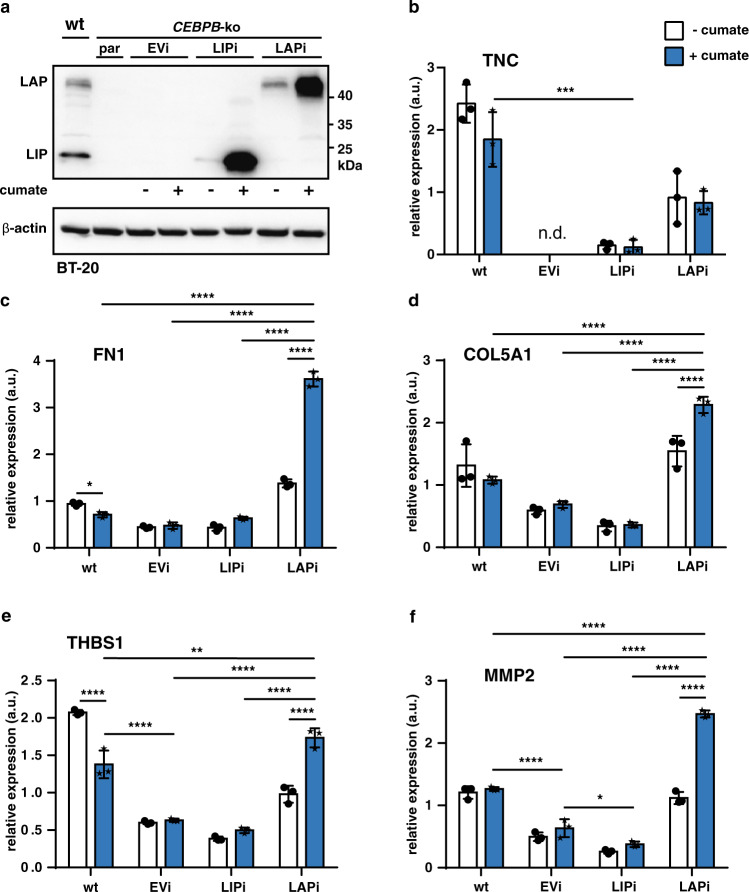
LAP induces the transcription of ECM components. **a** Immunoblot showing the expression of LAP and LIP and β-actin as loading control in BT-20 *CEBPB*-ko clone #7 transduced with expression vectors for cumate-inducible LIP and LAP expression (*n* = 3). **b**-**f** Expression of extracellular matrix-associated genes in *CEBPB*-ko clone #7 upon cumate-induced LIP and LAP expression relative to GAPDH. Differences analysed using a two-way ANOVA analysis and Tukey’s multiple comparison test. Error bars represent SD, *p < 0.05, **p < 0.01, ***p < 0.001 and ****p < 0.0001.

**Fig. 8:**
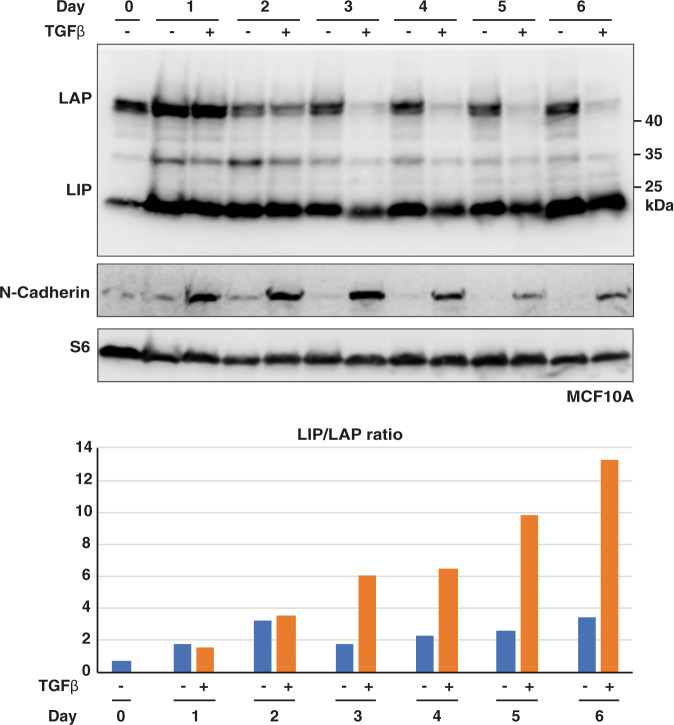
Treatment with TGFβ induces high LIP/LAP expression ratios. The upper immunoblot shows expression of LIP and LAP, the blot below the induction of the EMT marker N-cadherin. The lower blot shows expression of the ribosomal protein S6 for loading control. The bar graph show quantifications of relative LIP/LAP expression ratios.

The ECM is involved in cancer progression and metastasis at several levels ranging from the EMT to engraftment and outgrowth at distant sites^32–34^. To examine whether LIP or LAP alters expression of the ECM-related genes Tenascin (TNC), Collagen Type V alpha 1 (COL5A1), Fibronectin 1 (FN1), Thrombospondin 1 (THBS1) and (Matrix Metallopeptidase 2 (MMP2) we used the BT-20 system (Fig. 7a). *CEBPB*-ko in BT-20 cells results in downregulation of all five genes (Fig. 6a), suggesting that either LIP and/or LAP contribute to their activation. Re-expression of LIP in BT-20 *CEBPB*-ko cells does not alter the levels of the transcripts compared to the levels in the parent cells, suggesting that in absence of LAP, LIP does not further reduce the expression (Fig. 7b). Re-expression of LAP, however, strongly induces all five ECM transcripts. Note that the uninduced (- cumate) cells, because of some “leaky” induction of LAP, already show elevated LAP levels compared to EV control (Fig. 7a) and concomitant higher ECM transcript levels. These results indicate that in wt BT-20 cells the low levels of LAP relative to LIP (high LIP/LAP ratio) (Fig. 7a) is sufficient and required to activate ECM genes, which is beneficial for tumour progression and metastasis. Finally, to investigate whether the LIP/LAP ratio changes upon inducers of EMT we treated MCF10A cells with TGFβ which is known to induce EMT in these cells^35^. The induction of EMT was monitored by the upregulation of N-cadherin which was accompanied by a strong and permanent decrease of LAP levels and a slight and transient decrease in LIP levels resulting in a strong increase of the LIP/LAP ratio at the end of the treatment period (Fig. 8). These data support the hypothesis that a high LIP to LAP ratio is involved in the EMT process.

## Discussion

TNBC is an aggressive type of breast cancer characterised by the lack of expression of the hormone receptors ER and PR and the growth factor receptor HER2. These receptors are used as therapeutic targets for breast cancer types proficient for at least one of these factors. Their absence in TNBC makes treatment far more difficult and contributes to the poorer prognosis. Thus, a better understanding of other factors involved in TNBC is essential for the development of more effective therapies. In this study, we provide evidence that the expression ratio of the C/EBPβ protein isoforms LIP and LAP determines cell migration and cell invasion behaviour and contributes to the regulation of EMT- and ECM-related genes. We show that reverting the high LIP/LAP expression ratios typical for TNBC cells into low LIP/LAP ratios by ectopic overexpression of LAP inhibits cell migration and invasion. In addition, we show that overexpression of LIP promotes cell migration in MCF10A mammary epithelial cells or in *Cebpb*-deficient MEFs, suggesting that LIP in general supports cell migration. Breast cancer metastasis is a multistep process by which cells have to migrate, invade, extravasate, intravasate and colonise at distant target sites. Dissemination of tumour cells from the primary tumour requires cells to lose polarity and become migratory and invade the surrounding basement membrane and ECM. Initial steps of migration often require cells to lose adhesion molecules and epithelial markers and gain mesenchymal markers, in a process called EMT^29,30^. A few studies have addressed the role of C/EBPβ in breast cancer development and cancer cell migration, however, the data on isoform-specific roles are sparse and partially contradictory. For example, it was demonstrated that LIP supports anchorage-independent growth of breast epithelial cells through suppression of anoikis, a form of programmed cell death that occurs in anchorage-dependent cells when they detach from the surrounding ECM, which is a prerequisite for metastasis formation^36^. LIP was furthermore shown to upregulate the expression of the chemokine receptor CXCR4 and the pro-invasive CDH3/P cadherin in breast cancer cells, both known drivers of metastasis^5,37^. In addition, it was demonstrated that overexpression of LIP in the SCp2 mouse mammary epithelial cell line drives EMT using in vitro assays^38^. The downregulation of all C/EBPβ isoforms through microRNA-155 was shown to induce an EMT phenotype and migration and invasion in mouse mammary epithelial cells through a pathway that involves TGFβ signalling^39^. This observation is in line with another publication showing that shRNA-mediated downregulation of C/EBPβ expression induces the formation of metastasis in a mouse 4T1 mammary carcinoma cell line transplantation model^40^. The cell types in the latter two studies express low LIP/LAP ratios and the complete knockout or suppression of C/EBPβ may largely reveal effects through loss of the anti-migratory functions of LAP. In contrast, it was demonstrated that LAP induces the EMT and an invasive phenotype when overexpressed in untransformed MCF10A mammary epithelial cells through relocation of E-cadherin to the nucleus, mimicking loss of E-cadherin function^41^. The reason of these partially contradictory findings is not known, yet our data from migration and invasion assays support a pro-migratory role of LIP and suggest an anti-migration and anti-invasion function for the LAP isoform. The observation that the LIP/LAP ratio strongly increases upon TGFβ-induced EMT in MCF10A cells clearly points to an important role of LIP in the EMT process. In further support of a pro-migratory function of LIP is that we observed an increased migration of MCF10A cells by exogenous LIP expression, which is accompanied by a downregulation of E-cadherin and an upregulation of the mesenchymal marker N-cadherin and the migration and invasion promoting ECM protein Fibronectin, but not of the mesenchymal marker Vimentin and the ECM remodelling metalloprotease MMP2, suggesting a partial induction of EMT upon LIP overexpression. Furthermore, GSEA of the differentially regulated genes in the BT-20 *CEBPB*-ko cells revealed a mix of upregulated and downregulated EMT-related genes. This suggests that LIP may contribute to the EMT process likely in collaboration with other factors. Recent studies pointed out a greater plasticity between the epithelial and mesenchymal states, where cancer cells are observed to be in a spectrum of intermediary phases^32–34,42^. Single-cell migration often favours complete EMT to escape the tumour and detach from surrounding epithelial cells, whereas collective cell migration often is characterised by cells undergoing a partial EMT where the cancer cells retain some capacity of cell-cell adhesion and migrate in a collective manner^42^. This collective cell migration is often found in epithelial cancers, where the leader cells acquire a more mesenchymal phenotype by becoming motile and breaking down the surrounding ECM, leading the way for the follower cells^43^. Although only to be clarified by future studies, this may explain the mix of upregulated and downregulated EMT-genes upon modulation of LIP expression in our analyses. Studies have revealed an essential role of the ECM in tumour initiation, metastasis and the immune responses^33,44,45^. It has been reported that HER2-positive and TNBC tumours show an increased collagen content and ECM stiffness compared to luminal A and luminal B subtypes and that this is linked with the increased attraction of tumour-associated macrophages, TGFβ signalling and worse prognosis^46^. Although cancer-associated fibroblasts (CAFs) are the main producers of ECM, several studies have shown that interference with ECM production in cancer cells results in a significant delay in tumour progression. The ECM not only directs the cell migration by providing directional cues, but also provides chemical and physical signals to the leader cells, changing their migratory behaviour^47,48^. For example, it was reported that breast cancer cells produce Tenascin (TNC) as a metastatic niche component to prime to the lungs for breast cancer metastasis^49^. The matrix metallopeptidase 2 (MMP2) that is known to break down the basement membrane was shown to promote cancer invasion^50,51^. Fibronectin 1 (FN1) was shown to be expressed by leading invasive cancer cells and was linked to poor metastasis-free and overall survival in breast cancer^52,53^. Circulating Thrombospondin 1 (THBS1) is a marker for aggressive breast cancer^54^ and exposure to stromal THBS1 promotes breast cancer progression^55^. Our GSEA revealed a downregulation of ECM-related genes upon *CEBPB*-knockout in BT-20 cells, and LAP re-expression in BT-20 *CEBPB*-ko cells results in upregulation of pro-metastatic factors FN1, TNC, THBS1, COL5A1 and MMP2. Although this seems at odds with the inhibition of cell migration and invasion by LAP it points to a possible function of low LAP levels in transcriptional activation of these pro-metastatic genes. These results suggest that exclusively considering LIP as a competitive inhibitor of LAP maybe too simple and that we have to differentiate between LIP and LAP functions in tumour biology as Spike and Rosen describe in their recent review “C/EBPβ isoform-specific regulation is more complicated than we may think”^24^. LAP and LIP can dimerise with all available C/EBPα-ζ proteins in the cell with variable effects on target gene regulation. In addition, they have been shown to interact with other transcription factors like for example NFκB^56,57^, c-Jun^6^ or YY1^11,58^, further diversifying C/EBPβ functions. Nevertheless, there is strong and accumulating evidence that C/EBPβ-LAP and -LIP specific regulation is crucially involved in the development and/or progression of TNBC, and therefore a better understanding of their oncogenic functions is important in order to address potential suitability as a pharmacological target. Previously, we demonstrated in a screening approach using FDA-approved drugs that the LIP/LAP ratio is druggable also apart from known mTORC1 inhibitors^59^. Therefore, the LIP/LAP ratio could potentially be an interesting clinical target in TNBC. Taken together, we provide evidence that the LIP/LAP ratio regulates a subset of EMT and ECM-related genes and the migration/invasion potential of breast cancer cells. The presented study is limited by the lack of in vivo data showing LIP/LAP functions in breast tumour growth and metastasis. Future studies therefore should involve experiments using breast cancer mouse models with additional mutations altering expression of LIP and LAP separately in tumour cells.

## Methods

### Cell culture

TNBC cell lines BT-20, BT-549, MDA-MB-231, MDA-MB-468, CAL-120 and Hs 578T were cultured in DMEM (Gibco) supplemented with 10% FBS, 10 mM HEPES, 1 mM Sodium Pyruvate and 100 U/ml Penicillin Streptomycin. Hs 578 T were additionally supplemented with insulin (10 μg/ml). TNBC cell lines HCC1806 and HCC1143, luminal A breast cancer cell lines T-47D, MCF-7 and ZR-75-1 and the HER2-positive breast cancer cell lines BT-474, SK-BR-3 and ZR-75-30 were maintained in RPMI1640 medium (Gibco) supplemented with 10% FBS, 25 mM HEPES, 1 mM Sodium Pyruvate and 100 U/ml Penicillin/Streptomycin. The HER2-positive breast cancer cell line MDA-MB-361 was cultured in DMEM/HAM medium (Gibco) supplemented with 10% FBS, 10 mM HEPES, 1 mM Sodium Pyruvate and 100 U/ml Penicillin Streptomycin. MCF10A cells were cultured in DMEM/F12 containing 5% horse serum, 100 U/ml Penicillin/Streptomycin, supplemented with 30 ng/ml EGF, 100 ng/ml Cholera toxin, 10 μg/ml insulin, 0.5 mg/ml hydrocortisone, but cultured without EGF supplementation when used in migration experiments. To induce EMT, MCF10A cells were seeded to reach a confluency of 70–80% at day 0 and then treated with human recombinant TGFβ (4.0 ng/ml) for up to 6 days. During this period the medium containing the supplements mentioned above and TGFβ was renewed every second day while control cells received fresh medium with supplements but without TGFβ. For hormone treatments, MCF-7 cells were grown in phenol red-free RPMI medium with 10% charcoal-stripped FBS starting already 48 h before hormone supplementation. Cells were seeded in a concentration of 2.5 × 10^6^ cells per 5 cm plate and 40 h later (timepoint 0) β-estradiol (100 nM) or progesterone (100 nM) or the solvent control (ethanol) were added and the cells were cultivated further for up to 48 h. The cultivation of *Cebpb*^*ΔuORF*^ and corresponding wt MEFs, *Cebpb*-ko MEFs and *Cebpb*-ko MEFs ectopically expressing LIP was described before^28^. Induction with cumate was done for 48 h prior to start of the experiment using 10.000× cumate solution (BioCat 10.000× cat# PBQM100A-1, diluted to 1000× solution in EtOH and diluted 1:1000 in culture medium). All cell lines were incubated at 37 °C with 5% CO_2_.

### Cell lines

For generation of cell lines stably expressing cumate-inducible C/EBPβ-LIP and C/EBPβ-LAP constructs or an EV construct, HEK293T cells were transfected for lentivirus production. 4.5 × 10^6^ HEK293T cells were plated in 10-cm culture dishes. Twenty-four hours later, transfection was performed using the calcium phosphate method. The next morning, medium was changed and the morning thereafter, virus was harvested and BT-20 cells and BT-549 cells were infected with a cumate-inducible human C/EBPβ-LIP and C/EBPβ-LAP constructs or an EV construct using a standard protocol. Two days after infections cells were selected with puromycin 1 µg/ml for BT-20 and 0.5 µg/ml for BT-549. For overexpression of LIP in MCF10A cells, cells were infected with PLVX-IRES-Neo containing human C/EBPβ-LIP and selected using 1 mg/ml G418.

### DNA constructs

For overexpression of human C/EBPβ-LIP and C/EBPβ-LAP, the coding sequence was amplified from MCF-7 genomic DNA and the start codon of LAP was changed to an optimal Kozak sequence (ATCCATGGAAGTG to AGCCATGGAAGTG). The amplified PCR product was cloned into the pCDH-CuO-MCS-IRES-GFP-EF1α-CymR-T2A-Puro SparQTM All-in-one Cloning and Expression Lentivector (QM812B-1) (Invitrogen) and checked for mutations by Sanger sequencing. For overexpression of LIP in MCF10A cells, the human LIP sequence was amplified from MCF-7 genomic DNA and cloned into the PLVX-IRES-Neo vector.

### CRISPR/Cas9 gene targeting

gRNAs targeting human LIP and LAP were generated (gRNA 1 5′-AGTGGCCAACTTCTACTACG-3′, gRNA2 5′-CGCTTACCTCGGCTACCAGG-3′) and cloned into pSpCas9(BB)-2A-puro (PX459) v2.0 (http://www.addgene.org/62988/). Two days after electroporation (Lonza, Fugene Nucleofector Kit, protocol T-20), cells were selected with 1 μg/ml puromycin and seeded into clones. Several clones were isolated and the genomic *CEBPB* sequence was checked by Sanger sequencing. The absence of C**/**EBP**β** protein isoforms expression was confirmed by western blot.

### qRT-PCR analysis

mRNA was isolated using the RNeasy Isolation Kit (Qiagen) following the manufacture’s protocol. Primers for Tenascin C, Collagen5A1, MMP2, Fibronectin1, Thrombospondin1, E-cadherin, N-cadherin, and Vimentin were obtained via primerbank (https://pga.mgh.harvard.edu/primerbank) and listed below. Primers for GAPDH and were used for normalization. For the analysis of mRNA expression, 1 µg RNA was reverse transcribed with random hexamer primers using the transcriptor first-strand cDNA synthesis kit (Roche). qRT-PCR was preformed using the LightCycler 480 SYBR Green I Master Mix (Roche). qRT-PCR primer sequences used: FN1 forward CGGTGGCTGTCAGTCAAAG and reversed AAACCTCGGCTTCCTCCATAA; THBS1 forward AGACTCCGCATCGCAAAGG and reversed TCACCACGTTGTTGTCAAGGG; TNC forward TCCCAGTGTTCGGTGGATCT and reversed TTGATGCGATGTGTGAAGACA; MMP2 forward CCCCAAAACGGACAAAGAG and reversed CACGAGCAAAGGCATCATCC; COL5A1 forward GCCCGGATGTCGCTTACAG and reversed AAATGCAGACGCAGGGTACAG; E-Cadherin forward CGAGAGCTACACGTTCACGG and reversed AAATGCAGACGCAGGGTACAG; N-Cadherin forward TCAGGCGTCTGTAGAGGCTT and reversed ATGCACATCCTTCGATAAGACTG; Vimentin forward GACGCCATCAACACCGAGTT and reversed CTTTGTCGTTGGTTAGCTGGT; GAPDH forward TCAACGGATTTGGTCGTATTG and reversed TCTCGCTCCTGGAAGATGG.

### Immunoblot analysis

For protein extraction, the cells were washed twice with ice-cold 1 × PBS and lysed in 50 mM Tris pH 7.5, 150 mM NaCl, 1 mM EDTA, 1% Triton X-100, supplemented with protease and phosphatase inhibitors followed by sonication. Equal amounts of total protein were separated by SDS–PAGE, transferred to a PVDF membrane using either the Trans-Blot Turbo System (#170-4273, BIO-RAD) following the manufacturer’s protocol or tank blotting in 2.5 mM TRIS base, 20 mM glycine, 15% methanol. The following antibodies were used for detection: C/EBPβ (E299, ab32358; 1:1000 dilution), Tenascin C (EPR4219, ab108930; 1:1000 dilution) and Collagen V (ab7046; 1:1000 dilution) from Abcam, N-Cadherin (D4R1H, #13116; 1:1000 dilution) and Cyclin D1 (E3P5S, #55506; 1:1000 dilution) from Cell Signaling, Thrombospondin (A6.1, MA5-13398; 1:100 dilution) from Invitrogen, Fibronectin (NBP1-91258; 1:1000 dilution) from NOVUS Biologicals, Vinculin (hVIN-1, V9131; 1:20,000 dilution) from Merck, β-actin (clone C4, #691001; 1:10,000 dilution) from MP Biomedicals and α-tubulin from GeneTex (GTX112141; 1:10,000 dilution). For detection, HRP-conjugated secondary antibodies (Amersham Life Technologies, anti-rabbit NA934-1ML, anti-mouse NA931-ML; 1:5000 dilution) were used. The signals were visualised by chemiluminescence (Lightning Plus ECL reagent, Perkin Elmer or ECL prime reagent, Amersham Life Technologies) using the ImageQuant LAS 4000 mini imaging machine (GE Healthcare Bioscience AB) and the supplied software was used for quantification of the bands. All gels and immunoblots belonging to a single experiment are derived from this experiment and processed in parallel.

### Cellular migration assays

Prior to seeding for Incucyte scratch migration and invasion assays, cells were serum starved (in 1% serum). Cell migration and invasion was live monitored in the Incucyte Zoom using 96-well imagelock plates. BT-20, BT-549 and MCF10A cells were seeded at densities of 35,000 cells/well, 20,000 cells/well, and 20,000 cells/well respectively. Twelve hours post seeding a scratch wound was induced using the woundmaker, followed by washing of cells and incubation in low-serum medium. Cells were imaged every 2 h and using the incucyte zoom2018 software the Relative Wound Density was calculated. For Incuyte scratch invasion assays, wells were coated with 50 μl of 100 μg/ml matrigel (growth factor reduced, Corning # 354230) diluted in medium and embedded in 1 mg/ml matrigel in medium. For the MEF wound healing assay, the backside of each well of a six-well plate was marked with three lines. 2 × 10^6^ cells were seeded in prepared six-well plates 24 h prior to the assay. To induce a wound in the cell monolayer, tips of a 200 µl pipet were used to scratch in a 90° angle to the markings along the plate. Cells were washed once with PBS and medium was replaced. Pictures were taken every 4 h at the intersection between the scratch and the drawn line for 28 h. Pictures were quantified with imageJ software.

### Transwell cellular invasion assays

Prior to seeding for migration and invasion assays, cells were serum starved (in 1% FBS). Cells were seeded at densities of 20,000 cells/well in serum containing 1% medium in transwell permeable supports (6.5 mm insert, 8 μm polycarbonate membrane, corning #4322) and attracted to medium containing 10% FBS in the lower compartment. After 24 or 42 h, cells were fixed in 4% PFA and stained using 0.5% crystal violet. For imaging, three random images using the 5× objective were taken and average scores were plotted. For invasion assays, transwells were coated with 250 μg/ml matrigel in medium containing 1% FBS.

### Transcriptome analysis

RNA was isolated from three pools of BT-20 WT cells and three BT-20 *CEBPB*-ko clones, using the RNeasy Plus Mini Kit (QIAGEN). Quality and quantity of RNA was determined using Agilent’s Bioanalyzer 2100 in combination with the RNA 6000 nano chip (Agilent). Library preparation was done using Illumina’s TruSeq RNA v2 kit following the manufacturer’s description. The libraries were quality checked and quantified using Bioanalyzer 2100 in combination with the DNA 7500 kit (Agilent). Sequencing was done on a NextSeq500 in 75 bp, single-end sequencing, high-output mode. Sequence information was extracted using bcl2fastq v1.8.3 (Illumina), Sequencing resulted in around 50 million reads per sample. The quality of the reads was checked using FastQC (v. 0.11) and filtering and trimming of low-quality reads were performed using Trimmomatic (v. 0.33). Trimmed reads were aligned to the GRCh38/hg38 genome (genome annotation from ensemble release 92) using STAR aligner v. 2.6.0b^60^. Identification of differentially expressed genes (DEGs) was done using the R package DESeq2^27^. The resulting p-values were adjusted using Benjamini and Hochberg’s approach for controlling the false discovery rate^61^. Genes were regarded as differentially expressed if adjusted *p*-values were <0.05. Functional clustering analysis was performed using the DAVID database (https://david.ncifcrf.gov/, version 6.7) at default settings with medium stringency (downregulated genes Fig. 2d) or highest stringency (Fig. S1A). GSEA was performed using the GSEA desktop application and compared to the Hallmark gene sets. The phenotype label was set as *CEBPB* WT vs *CEBPB*-ko. The t-statistic mean of the genes was computed for each hallmark gene set using a permutation test with 1000 replications.

### Statistics and reproducibility

For analysis of differentially expressed genes (transcriptome) we refer to the specific sections above. Other statistical differences were calculated by Student’s *t*-test or two-way ANOVA as indicated using GraphPad Prism 8. Data are presented as the mean ± standard deviation (SD). Statistically significant differences are indicated with *p < 0.05, **p < 0.01, ***p < 0.001.

### Reporting summary

Further information on research design is available in the Nature Research Reporting Summary linked to this article.

## Supplementary information

Supplementary Information

Reporting Summary

Supplementary Data 1

## Data Availability

The RNA-seq data is deposited to ArrayExpress (https://www.ebi.ac.uk/arrayexpress/) under accession number E-MTAB-11227, and at figshare 10.6084/m9.figshare.16988212.v1. The analysed data are available as Supplementary Data 1.
