# Supplementary material for: C/EBPβ isoform-specific regulation of migration and invasion in triple-negative breast cancer cells
Source: NPJ Breast Cancer. 2022 Jan 18;8:11. doi: 10.1038/s41523-021-00372-z (PMC8766495; doi:10.1038/s41523-021-00372-z)
Supplement: Supplementary file 1 — Supplementary Information [file 41523_2021_372_MOESM1_ESM.pdf]

Supplemenatry Figures 1-4 and Table 1

C/EBPβ isoform-specific regulation of migration and invasion in triple-negative breast cancer cells

Britt A. Sterken[1], Tobias Ackermann[1$], Christine Müller[1$], Hidde R. Zuidhof[1], Gertrud Kortman[1], Alejandra Hernandez-Segura[1], Mathilde Broekhuis[2], Diana Spierings[3], Victor Guryev[1], Cornelis F. Calkhoven[1]*

[1]European Research Institute for the Biology of Ageing (ERIBA), [2]iPSC CRISPR facility, [3]Research Sequencing facility, University Medical Center Groningen, University of Groningen, 9700 AD Groningen, The Netherlands.

[$]These authors contributed equally

*corresponding author: c.f.calkhoven@umcg.nl

Supplementary Figure 1

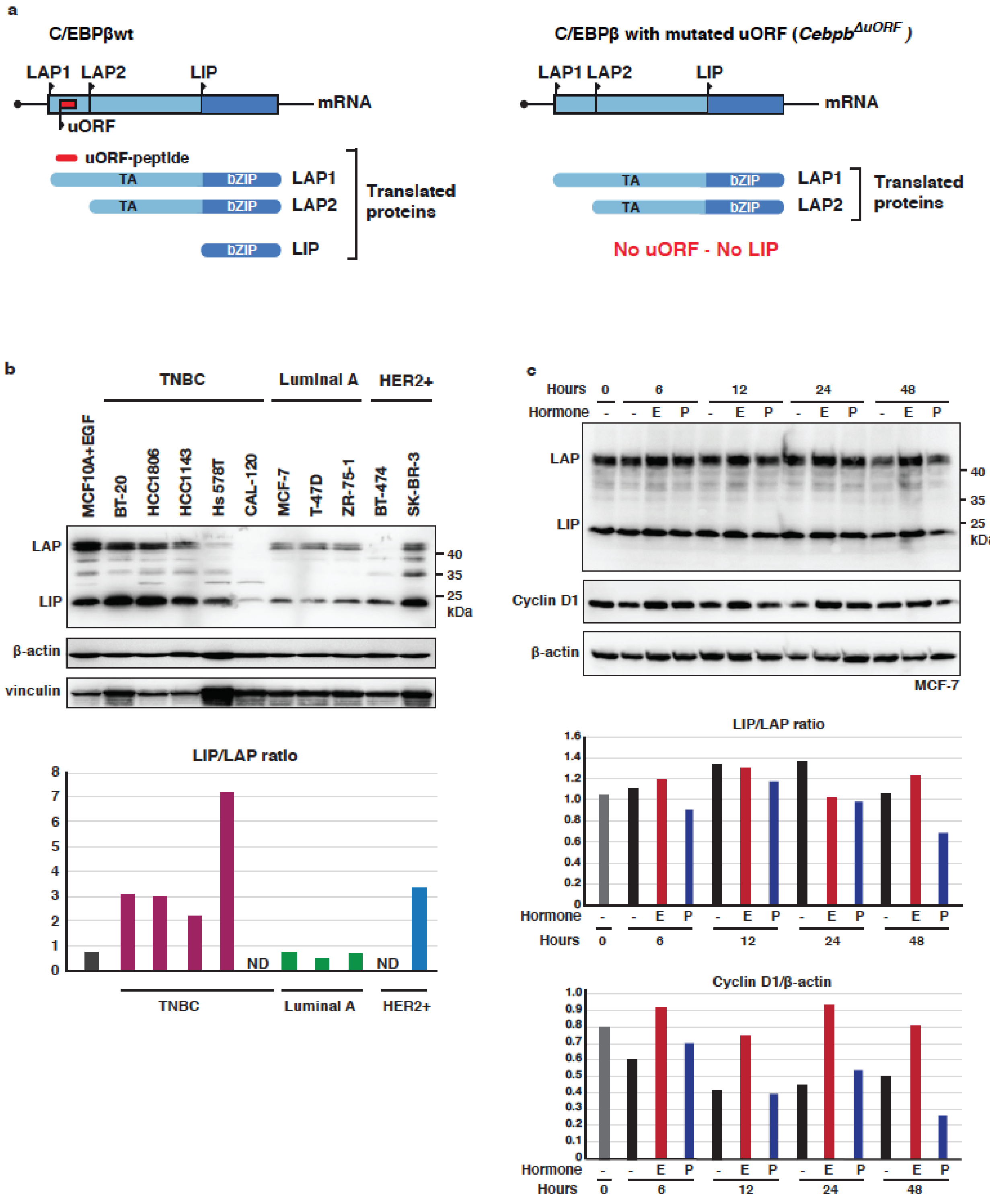

d

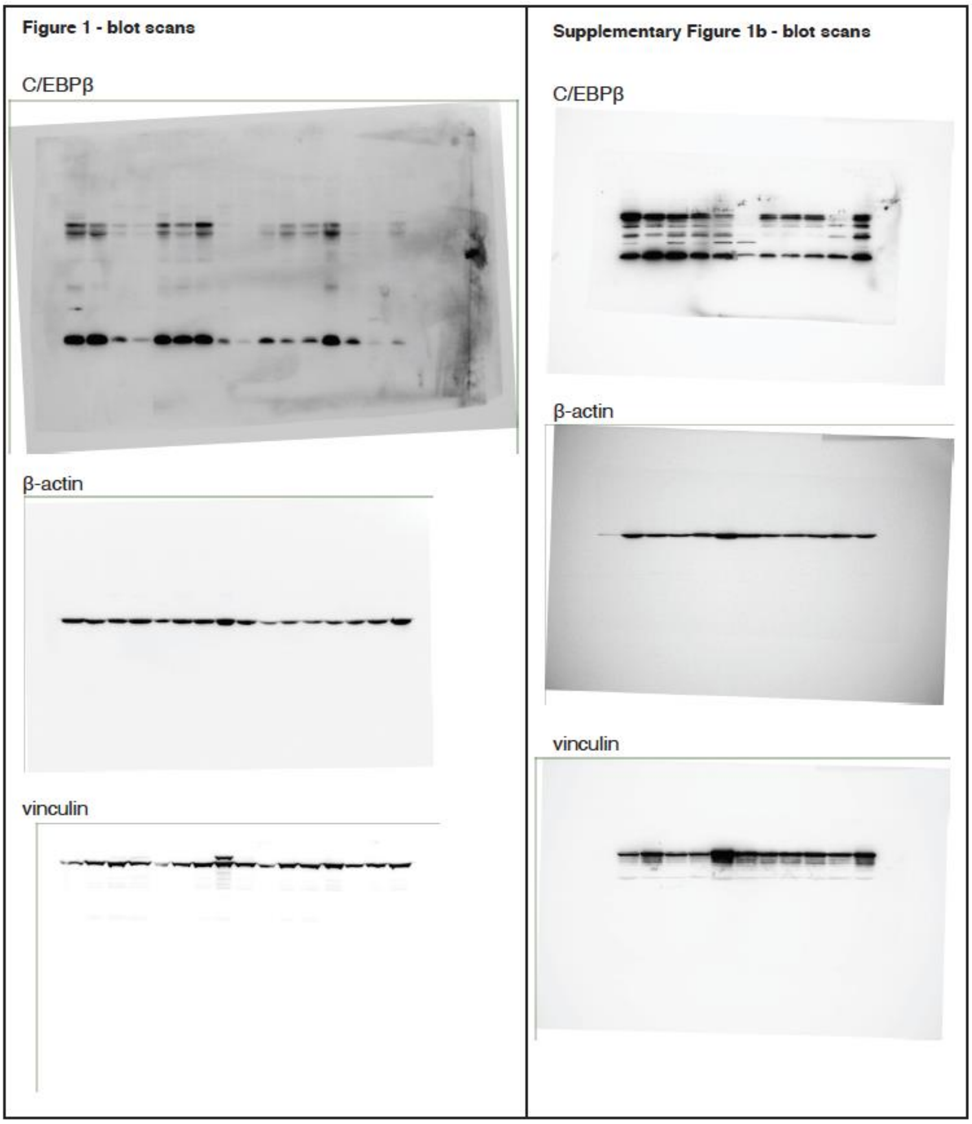

Supplementary Figure 1. (**a**) Schematic representation of the C/EBPβ protein isoform expression for wt CEBPB-mRNA or CEBPB-mRNA with mutated uORF (*CEBPB*$^{\Delta uORF}$)[1]. bZIP: basic leucine zipper DNA-binding domain; TA: transactivation domains. (**b**) The immunoblot shows the expression of LIP and LAP in a panel of TNBC (ER-, PR-, HER2-), luminal A (ER+, PR+/-, HER2-), HER2-positive (+) (ER-, PR-, HER2+), luminal B (-B) (ER+, PR+/-, HER2+) type breast cancer cell lines[2] and in untransformed MCF10A supplemented with EGF required for proliferation and known to induced LIP. The blots below show β-actin and vinculin as loading controls. The bar graph shows the relative LIP/LAP isoform ratio quantification per cell line of the blot shown. The LAP signal of CAL-120 and BT-474 were too low for reliable quantification. (**c**) Upper immunoblot shows the expression of LIP and LAP in MCF-7 cells treated with either progesterone (P) or estrogen (E) or solvent (-). Middle blot shows CyclinD1 expression known to be induced by ER, and the lower blot shows β-actin expression as loading control. The bar graph show quantifications of relative LIP/LAP expression ratios or CyclinD1 expression levels related to β-actin control, respectively. (**d**) Original immunoblot scans for Fig.1 and Supplementary Fig. 1b.

Supplementary Figure 2

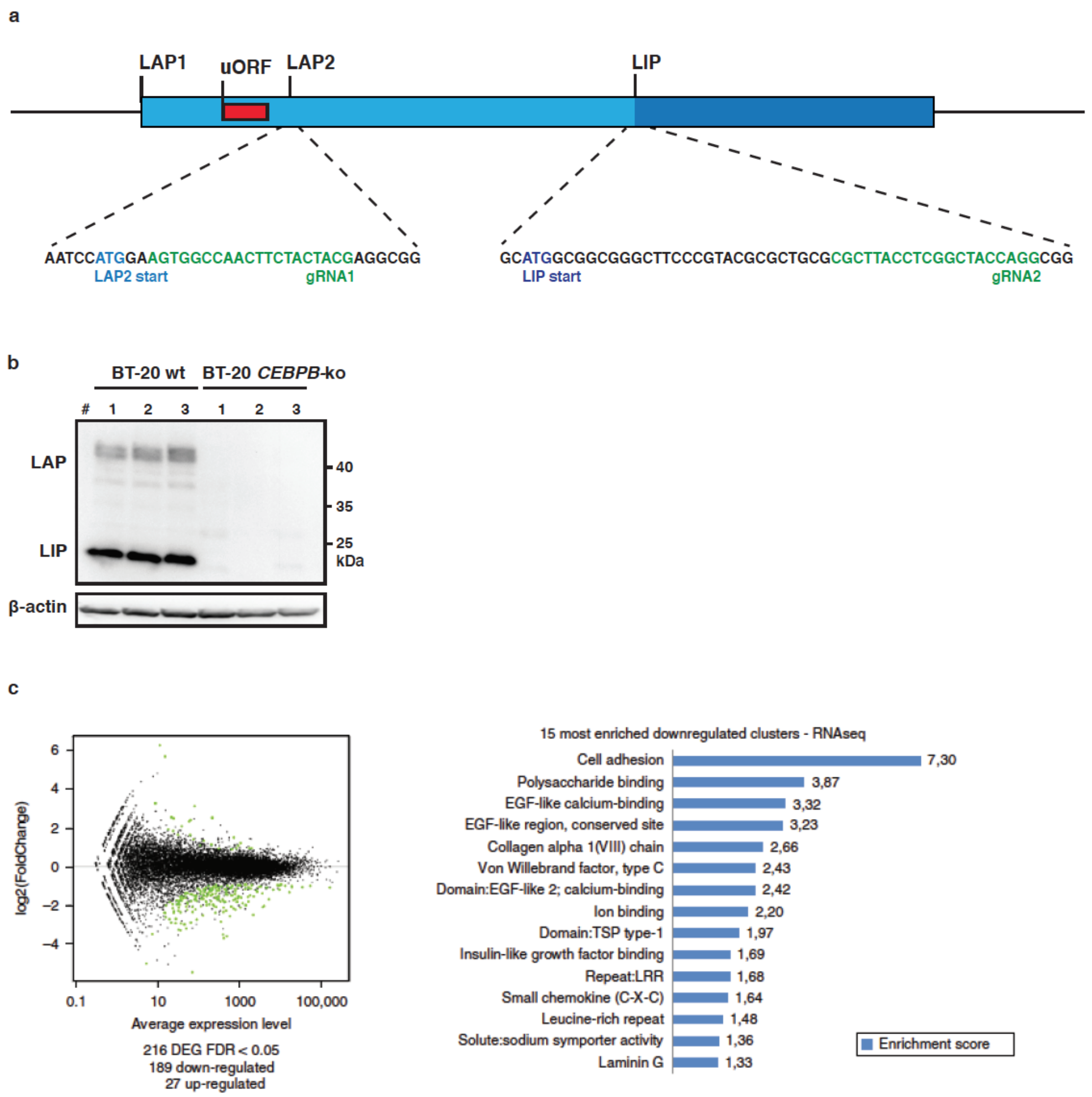

Supplementary Figure 2. (**a**) Schematic overview of the *CEBPB*-coding region and gRNAs used for CRISPR/Cas9 targeting for *CEBPB*-knockout. (**b**) Immunoblot for analysing LAP and LIP expression of three clones of BT-20 wt and BT-20 *CEBPB*-ko cells. β-actin was used as loading control. (**c**) Differential expressed genes (DEG) by ectopic expression of LIP in *Cebpb*-ko MEFs compared to empty vector control (EV) with an FDR < 0.05 and most enriched downregulated clusters (DAVID) (high stringency); data from Ackermann et al (2019) DOI:10.1038/s42003-019-0461-z.

Supplementary Figure 3

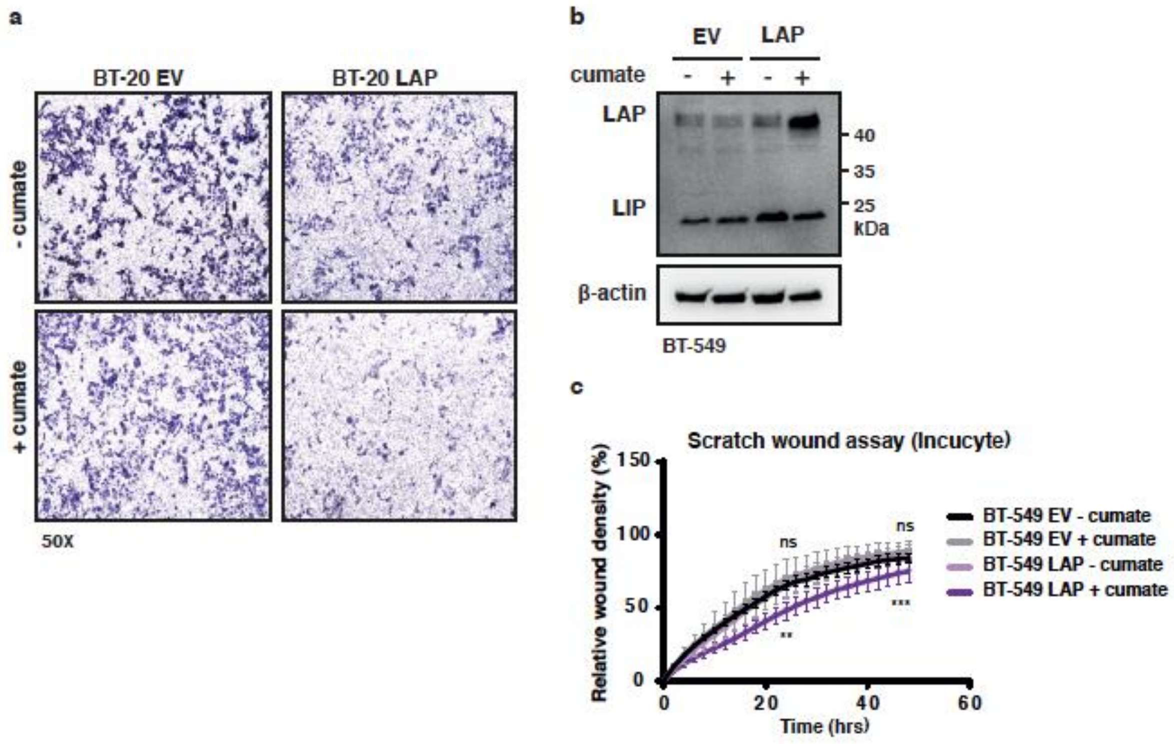

Supplementary Figure 3. (**a**) Representative images of Boyden chamber invasion assay of BT-20 cells containing cumate-inducible LAP expression construct or empty vector control (EV). (**b**) Immunoblot showing the expression of LAP and LIP in BT-549 cells containing a cumate-inducible LAP construct or empty vector control (EV). β-actin was used for loading control. (**c**) Time course of Incucyte scratch wound migration assay shown as relative wound density of BT-549 cells containing a cumate-inducible LAP construct or empty vector control induced with cumate (n=4). Statistical differences were analysed for timepoints 24 and 48 hrs using Two-way Anova analysis and Tukey's multiple comparisons test (BT-549 EV - cumate vs BT-549 EV + cumate were compared (ns), and BT-549 LAP - cumate vs BT-549 LAP + cumate were compared (**)). Error bars represent SD, * p<0.05, ** p<0.01, *** p<0. 001

Supplementary Figure 4

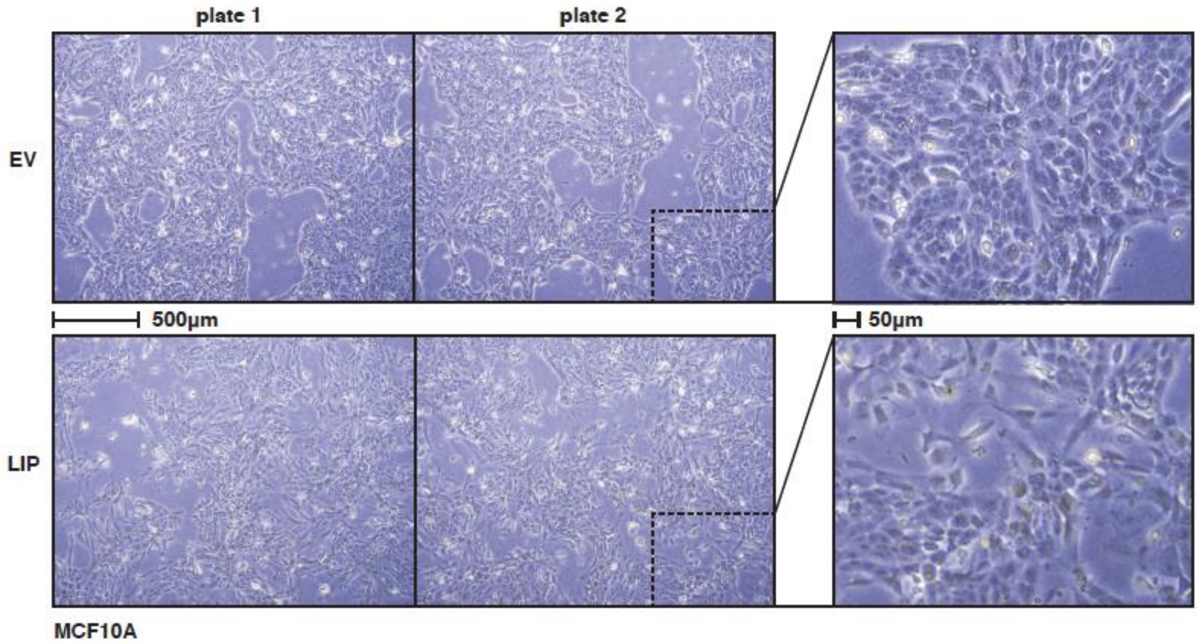

Supplementary Figure 4. Microscopic pictures of two cell culture plates with MCF10A cells transduced with either empty vector control (EV) or LIP expression vector (LIP). At the right a magnification of an area of plates 2 are shown.

Supplementary Table 1. C/EBPβ-associated fragments associated with H3K acetylation (H3K27Ac).

Gene	C/EBPβ site position	Cell line	Score (out of 1000)	Relative to TSS	Overlap H3K27ac
FN1	chr2:215417422-215417625	HepG2	296	18442	yes
FN1	chr2:215418620-215418878	A549, HepG2, HepG2, IMR-90	940	17189	no
FN1	chr2:215420637-215420860	HepG2	192	15207	yes
FN1	chr2:215436368-215436640	A549, HepG2, IMR-90, K562	1000	-573	yes
FN1	chr2:215451043-215451325	A549, HepG2, IMR-90, K562	768	-15258	no
THBS1	chr15:39579593-39579668	IMR-90	586	1486	yes
THBS1	chr15:39580651-39580906	A549, IMR-90	916	428	yes
THBS1	chr15:39574580-39574844	A549, IMR-90	285	6499	no
TNC	chr9:115132700-115132816	IMR-90	630	-14659	yes
TNC	chr9:115134999-115135254	IMR-90	333	-17097	yes
TNC	chr9:115135923-115136178	IMR-90	472	-18021	yes
TNC	chr9:115106029-115106257	A549, HepG2, IMR-90	1000	11900	yes
TNC	chr9:115107288-115107543	IMR-90	302	10614	no
TNC	chr9:115115966-115116211	A549, IMR-90	1000	1946	yes
MMP2	chr16:55480463-55480741	HepG2, IMR-90, K562	1000	-1265	yes
MMP2	chr16:55472618-55472742	IMR-90	599	6580	yes
COL5A1	chr9:134644738-134644964	A549, IMR-90	1000	-2935	yes
COL5A1	chr9:134649201-134649456	IMR-90	291	-7398	yes
COL5A1	chr9:134624062-134624317	A549	214	17741	yes
COL5A1	chr9:134624974-134625235	IMR-90	975	16829	yes
COL5A1	chr9:134626770-134627025	IMR-90	216	15033	No

Used: genome browser + ENCODE database (https://genome.ucsc.edu). Genome: Human GRCh38/hg38. Reported: binding sites of C/EBPβ 20 kB up-and downstream of the Transcription Start Site (TSS)

References

1 Müller, C. *et al.* Reduced expression of C/EBPbeta-LIP extends health and lifespan in mice. *Elife* **7**, doi:10.7554/eLife.34985 (2018).

2 Dai, X., Cheng, H., Bai, Z. & Li, J. Breast Cancer Cell Line Classification and Its Relevance with Breast Tumor Subtyping. *J Cancer* **8**, 3131-3141, doi:10.7150/jca.18457 (2017).
