# Supplementary material for: C/EBPβ isoform-specific regulation of migration and invasion in triple-negative breast cancer cells
Source: NPJ Breast Cancer. 2022 Jan 18;8:11. doi: 10.1038/s41523-021-00372-z (PMC8766495; doi:10.1038/s41523-021-00372-z)
Supplement: Supplementary file 2 — Reporting Summary [file 41523_2021_372_MOESM2_ESM.pdf]

nature portfolio

Corresponding author(s): Cornelis F. Calkhoven

Last updated by author(s): Nov 10, 2021

Reporting Summary

Nature Portfolio wishes to improve the reproducibility of the work that we publish. This form provides structure for consistency and transparency in reporting. For further information on Nature Portfolio policies, see our Editorial Policies and the Editorial Policy Checklist.

Statistics

For all statistical analyses, confirm that the following items are present in the figure legend, table legend, main text, or Methods section.

n/a	Confirmed	
☐	☒	The exact sample size (n) for each experimental group/condition, given as a discrete number and unit of measurement
☐	☒	A statement on whether measurements were taken from distinct samples or whether the same sample was measured repeatedly
☐	☒	The statistical test(s) used AND whether they are one- or two-sided<br>*Only common tests should be described solely by name; describe more complex techniques in the Methods section.*
☐	☒	A description of all covariates tested
☐	☒	A description of any assumptions or corrections, such as tests of normality and adjustment for multiple comparisons
☐	☒	A full description of the statistical parameters including central tendency (e.g. means) or other basic estimates (e.g. regression coefficient) AND variation (e.g. standard deviation) or associated estimates of uncertainty (e.g. confidence intervals)
☐	☒	For null hypothesis testing, the test statistic (e.g. F, t, r) with confidence intervals, effect sizes, degrees of freedom and P value noted<br>*Give P values as exact values whenever suitable.*
☒	☐	For Bayesian analysis, information on the choice of priors and Markov chain Monte Carlo settings
☒	☐	For hierarchical and complex designs, identification of the appropriate level for tests and full reporting of outcomes
☒	☐	Estimates of effect sizes (e.g. Cohen's d, Pearson's r), indicating how they were calculated

Our web collection on statistics for biologists contains articles on many of the points above.

Software and code

Policy information about availability of computer code

Data collection	ImageQuant TL 8.1, Incucyte S3, LightCycler® 480, Genome Browser + Encode database (http://genome.ucsc.edu/ENCODE/), HiSeq2500, bcl2fastq v1.8.3 (Illumina)
Data analysis	Microsoft Excel, LightCycler® 480 Software, incucyte zoom2018 software, GraphPad Prism 6, ImageJ, Encode database (http://genome.ucsc.edu/ENCODE/), DAVID functional clustering analysis (https://david.ncifcrf.gov/, version 6.7), GSEA desktop application, R with package DESeq, STAR aligner v. 2.6.0b

For manuscripts utilizing custom algorithms or software that are central to the research but not yet described in published literature, software must be made available to editors and reviewers. We strongly encourage code deposition in a community repository (e.g. GitHub). See the Nature Portfolio guidelines for submitting code & software for further information.

Data

Policy information about availability of data

All manuscripts must include a data availability statement. This statement should provide the following information, where applicable:

- Accession codes, unique identifiers, or web links for publicly available datasets
- A description of any restrictions on data availability
- For clinical datasets or third party data, please ensure that the statement adheres to our policy

All raw data will be made available on request. The RNA-sequencing data are in the Suplementary Table 1 and will be deposited in a public database.

Field-specific reporting

Please select the one below that is the best fit for your research. If you are not sure, read the appropriate sections before making your selection.

☒ Life sciences ☐ Behavioural & social sciences ☐ Ecological, evolutionary & environmental sciences

For a reference copy of the document with all sections, see nature.com/documents/nr-reporting-summary-flat.pdf

Life sciences study design

All studies must disclose on these points even when the disclosure is negative.

Sample size	No statistical method was used to determine the sample size. Most sample sizes were picked to allow statistical analysis.
Data exclusions	No data was excluded.
Replication	Most cell culture experiments were reproduced three times as specified in the figure legends.
Randomization	Randomization is not relevant to this study because effects of protein overexpression or downregulation were investigated. Therefore different cell lines had to be used. However, upon generation of the cell lines cells were not selected for special features.
Blinding	Investigators were not blinded to group allocation during the study. This was not possible since for most experiments the person who performed the experiment, collected the data and analyzed the data was the same.

Reporting for specific materials, systems and methods

We require information from authors about some types of materials, experimental systems and methods used in many studies. Here, indicate whether each material, system or method listed is relevant to your study. If you are not sure if a list item applies to your research, read the appropriate section before selecting a response.

Materials & experimental systems

n/a	Involved in the study	
☐	☒	Antibodies
☐	☒	Eukaryotic cell lines
☒	☐	Palaeontology and archaeology
☒	☐	Animals and other organisms
☒	☐	Human research participants
☒	☐	Clinical data
☒	☐	Dual use research of concern

Methods

n/a	Involved in the study	
☒	☐	ChIP-seq
☒	☐	Flow cytometry
☒	☐	MRI-based neuroimaging

Antibodies

Antibodies used	C/EBPβ (E299, ab32358), Tenascin C (EPR4219, ab 108930) and Collagen V (ab7046) from Abcam, N-Cadherin (D4R1H, #13116) and Cyclin D1 (E3P5S, #55506) from Cell Signaling, Thrombospondin (A6.1, MA5-13398) from Invitrogen, Fibronectin (NBP1-91258) from NOVUS Biologicals, Vinculin (hVIN-1, V9131) from Merck, β-actin (clone C4, #691001) from MP Biomedicals and α-tubulin from GeneTex (GTX112141). For detection, HRP-conjugated secondary antibodies, anti-rabbit NA934-1ML, anti-mouse NA931-ML (Amersham Life Technologies) were used.
Validation	All antibodies are validated by the supplying companies with information about used techniques available at the company's websites.

Eukaryotic cell lines

Policy information about cell lines

Cell line source(s)	ATCC and own lab for MEFs
Authentication	By microscopic morphology.
Mycoplasma contamination	The cell lines were tested for mycoplasma contamination.
Commonly misidentified lines (See ICLAC register)	None
